# Factors That Affect the COVID-19 Pandemic in Summer 2022 Compared to Summer 2021

**DOI:** 10.3390/ijerph191912561

**Published:** 2022-10-01

**Authors:** Marharyta Sobczak, Rafał Pawliczak

**Affiliations:** Department of Immunopathology, Division of Biomedical Science, Faculty of Medicine, Medical University of Lodz, 90-752 Lodz, Poland

**Keywords:** COVID-19, SARS-CoV-2, multiple factor analysis, Omicron, restrictions, vaccination

## Abstract

The COVID-19 pandemic still goes on. The increasing number of COVID-19 cases has been observed since the start of summer 2022, although this was not in summer 2021. Therefore, we would like to compare factors that were responsible for this trend in five selected countries in the European Union (Greece, Italy, Slovenia, Austria and Germany) using the data from publicly available databases for the analyzed period of weeks 22–30 in 2021 and 2022. The multiple factor analysis was conducted in R, using mean or median score. Our cross-sectional study showed that analyzed countries had similar profiles in 2021 characterized by restrictions and health system policies, as well as B.1.351, B.1.1.7, B.1.617.2 and P.1 variants. Similarly, these countries had similar profiles in 2022, but described by other variables: number of new COVID-19 cases per million, number people fully vaccinated per hundred, number of total boosters administered per hundred and also occurrence of Omicron variant and its sub-lineages. Although the COVID-19 vaccination rate is relatively high in the European Union, during the summer of 2022, the number of COVID-19 cases sharply increased daily, which seems to be connected with the presence of the Omicron variant and its sub-lineages.

## 1. Introduction

Since the beginning of the COVID-19 pandemic in December 2019, scientists from all around the world focused on stopping this disease. Of course, the most promising approach is the discovery of vaccines. However, in the early time of the pandemic, prevention was considered the most effective approach to control COVID-19. Most countries have introduced equal government restrictions, such as social distancing, wearing masks, staying home, quarantine, disinfecting and handwashing [1,2]. In addition, there were differences in society’s attitudes towards the pandemic, which are based on culture–for example in Asian and Western cultures [3]. Social distancing strategies can be divided into five categories, starting with restrictions, prohibitions and closures to incentives, and even punishments. In general, these strategies were focused on travel restriction, school closure, and the usage of virtual communication [1].

SARS-CoV-2, the virus that is responsible for COVID-19, is a single-strand positive-sense RNA virus [4]. Thanks to the genomic sequence monitoring of SARS-CoV-2, the evolution of this virus can be observed. Mutations in viral genomes led to the origin of many variants of the virus all over the world. According to transmission potential, these variants can be divided into few groups: variants of interest (VOI), variants of concern (VOC), and variants under monitoring (VUM) [5]. On the day of 11 August 2022, a currently circulating VOC is Omicron [6]. B.1.1.529 lineage has at least 32 mutations, including 15-point mutations in the receptor binding domain (RBD), insertions and deletions [7]. Moreover, this variant of SARS-CoV-2 has been divided into a few sub-lineages. BA.1 sub-lineage has 37 mutations in RBD and can infect the upper respiratory tract in comparison to Delta variant, which infects the lower respiratory tract. This can explain why BA.1 sub-lineage has the higher transmissibility but may cause less severe COVID-19. Later, in some countries, BA.2 sub-lineage quickly displaced the other lineages, such as Delta and BA.1 [8]. At the same time, another sub-lineage BA.3 appeared for the first time in South Africa and has 10 mutations originating from BA.1 and 2 mutations from BA.2, which affect the spike protein formation process [9]. Similarly, in South Africa, other sub-lineages of Omicron were detected, such as BA.4 and BA.5, which have evolved from the BA.2 sub-lineage. However, currently the BA.5 sub-variant is responsible for more COVID-19 infections than BA.4 [10].

As of 11 August 2022, 72.7% of the population have received the primary course of the vaccine in the European Union countries [11]. Regression analysis shows that the COVID-19 pandemic may decline in regard to COVID-19 mortality in 2022 [12]. Moreover, the comparative analysis of data from Italy between April–September 2020 and 2021 showed the seasonal behavior of COVID-19 irrespective of government restrictions as well as vaccination [13]. Even so, the increase of COVID-19 infections was observed in the summer of 2022. Therefore, the aim of our cross-sectional study was to compare the factors that affect the incidence of COVID-19 in the summer of 2021 and 2022, especially 22–30 weeks in five selected countries in the European Union. We assumed that the analyzed countries would be characterized by similar profiles in the analyzed time periods.

## 2. Materials and Methods

### 2.1. Data Search and Extraction

On the date of August 04, 2022, we chose five countries in the European Union with the most numbers of new COVID-19 cases in the last 7 days per million based on Worldometer [14], which was the criterium of countries selection. After this, we extracted data from European Centre for Disease Prevention and Control [15], Oxford COVID-19 Government Response Tracker [16], Our World in Data [17] and Eurocontrol [18] between weeks 22 and 30 in 2021 and 2022. From this period, we collected the data, such as:

- The number of new cases per million describes daily numbers of COVID-19 cases per million people;
- The number of cases with different variant of SARS-CoV-2 describes the weekly numbers of COVID-19 cases with different variants of SARS-CoV-2;
- The number of people fully vaccinated per hundred describes the number of people who received all vaccine doses per hundred people;
- The total boosters per hundred describes the number of people who received booster doses per hundred people;
- Number of flights describes the number of air traffic.

The number of cases with different variants of SARS-CoV-2 were calculated as the number of cases with different variants of SARS-CoV-2 per million using data from Our World in Data [17]. In addition to this, we extracted scaled data such as:

- School closing (scale 0–3) describes the school and university closing;
- Workplace closing (scale 0–3) describes workplace closing;
- Cancelation of public events (scale 0–2) describes public events cancelling;
- Restrictions on gatherings (scale 0–4) describes the limits on gatherings;
- Close public transport (scale 0–2) describes public transport closing;
- Stay at home requirements (scale 0–3) describes recommendations to “stay at home”;
- Restrictions on internal movement (scale 0–2) describes restrictions on internal travels between cities and regions;
- International travel controls (scale 0–4) describes restrictions on international travel;
- Testing policy (scale 0–3) describes the government policy on accessing of PCR testing;
- Contact tracing (scale 0–2) describes government policy on contact tracing after a positive COVID-19 diagnosis;
- Facial coverings (scale 0–4) describes government policy on the use of facial coverings;
- Vaccination policy (scale 0–5) describes government policy on vaccine availability for different groups;
- Protection of elderly people (scale 0–3) describes government policy on protecting elderly people.

### 2.2. Statistical Analysis

Using collected data, we conducted the multiple factor analysis (MFA), which is similar to principal component analysis (PCA) but used for different types of variables, such as categorical, quantitative and frequency. MFA consists of two steps: at first, it calculates the PCA and normalizes each data table, in which sets of variables are collected. In the second step, these data tables are combined into a joined data table that is analyzed by PCA [19,20]. We calculated the mean score for continuous variables and median for categorical variables. Additionally, for the continuous variable we used standardization and centered around zero. Variables were grouped as new cases, variants, vaccinations, flights, restrictions and health system policies as active groups, and years and countries names as supplementary groups. MFA analysis was conducted in R (version 4.2.1).

## 3. Results

### 3.1. Countries Selection

**Figure 1:**
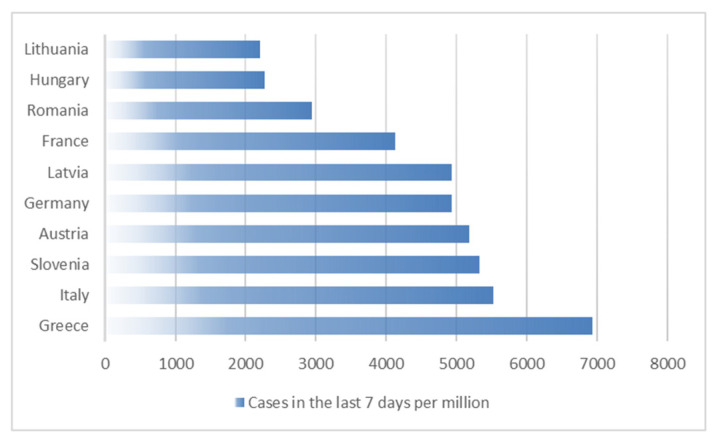
Top 10 countries in the European Union with the highest number of COVID-19 cases in the last 7 days per million as of 4 August 2022.

Figure 1 shows top 10 countries in the European Union (EU) with the higher number of COVID-19 cases in the last 7 days per million on the day 4 August 2022 based on data from Worldometer [14]. From this, we selected five countries with the highest value of the coefficient. These were: Greece, Italy, Slovenia, Austria and Germany.

### 3.2. The Distribution of SARS-CoV-2 Variants in 5 Selected Countries in the EU

**Figure 2:**
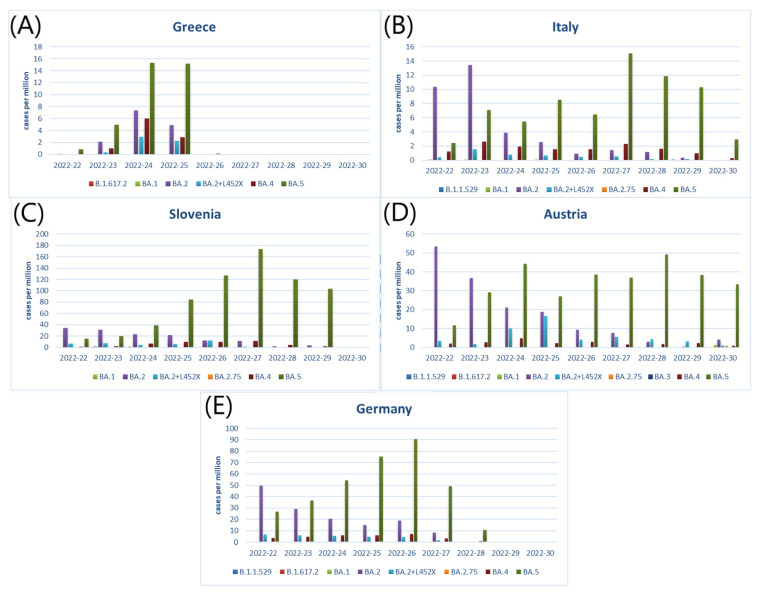
SARS-CoV-2 variants in (**A**) Greece, (**B**) Italy, (**C**) Slovenia, (**D**) Austria and (**E**) Germany in the period of weeks 22 to 30 2022.

Afterwards, we checked the distribution of SARS-CoV-2 variants in selected countries. As shown in Figure 2A–E, Omicron variant, especially sub-variant BA.5, was dominant among the COVID-19 cases during the analyzed period from 22 to 30 weeks of 2022.

### 3.3. Contributing Variables to the Dimensions Definition

**Figure 3:**
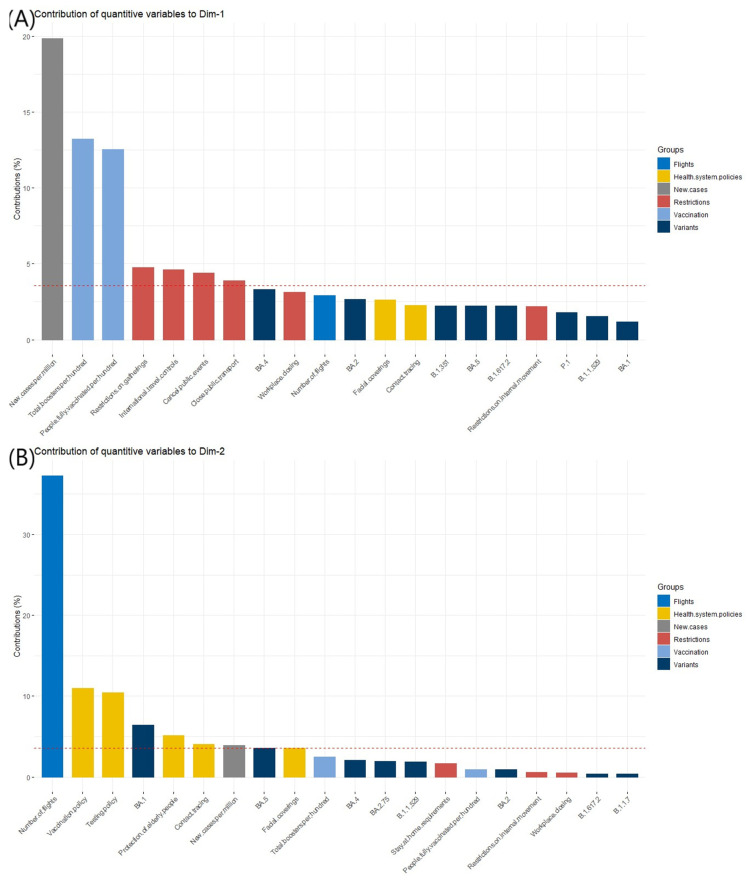
The contribution of quantitative variables to the definition of the dimensions. (**A**) contribution of quantitative variables to Dim-1; (**B**) contribution of quantitative variables to Dim-2.

In the next step, we discovered that ‘new cases per million’ was the most contributing variable to the definition of Dim-1 (dimension 1), whereas ‘number of flights’ to Dim-2 (dimension 2). These results were shown on Figure 3A,B.

### 3.4. The Relationships between Analyzed Variables

**Figure 4:**
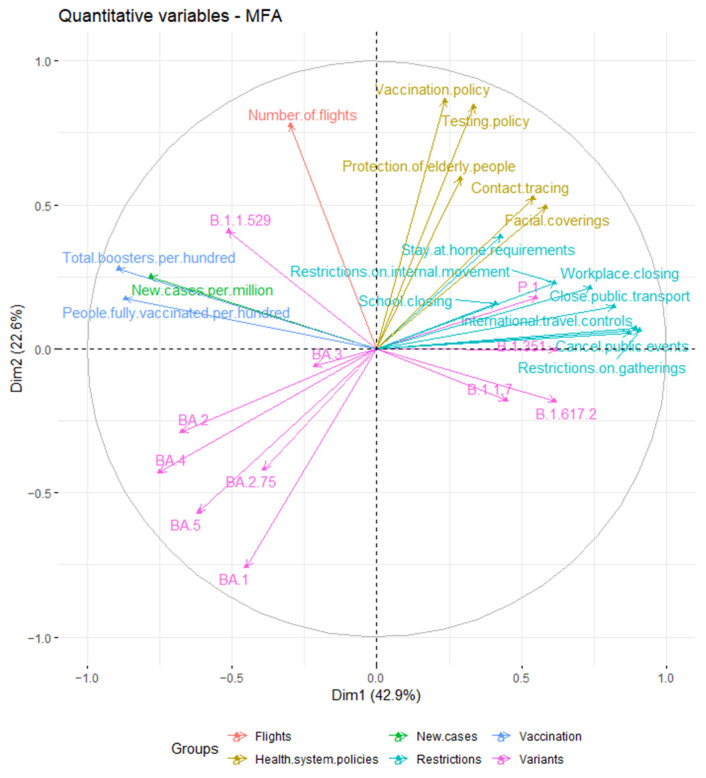
Scatter plot of analyzed variables.

A correlation plot (Figure 4) showed the relationships and correlation between analyzed variables. We observed that restrictions and health system policies groups were positively correlated. Moreover, SARS-CoV-2 variants such as P.1, B.1.351, B.1.1.7 and B.1.617.2 were also positively correlated with restrictions and health system policies groups. Simultaneously, the above-mentioned groups negative correlated with lineages of the Omicron variant (BA.1, BA.2, BA.2.75, BA.3, BA.4 and BA.5). On the other hand, the vaccination group, especially the numbers of total boosters per hundred, was strongly positively correlated with new cases of COVID-19 per million and Omicron lineages.

### 3.5. The Comparison of Countries Profiles in Summer 2022 and Summer 2021

**Figure 5:**
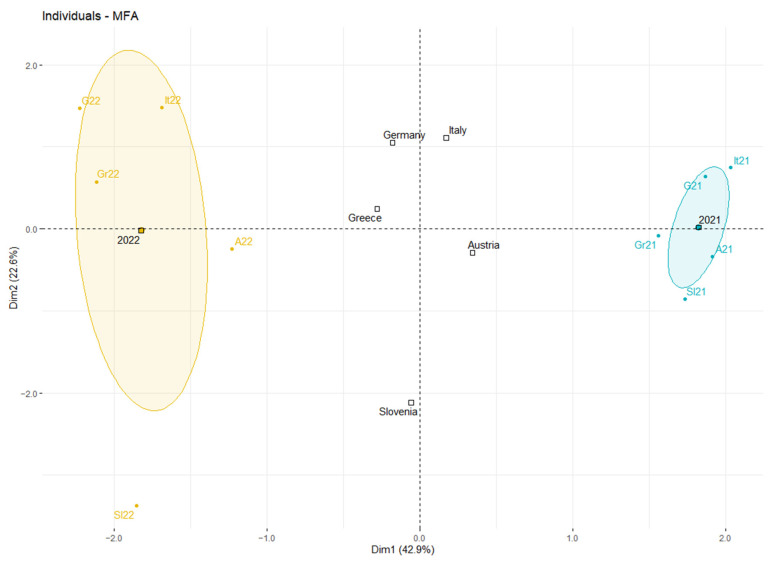
Individual results according to 2021 and 2022. It—Italy, G—Germany, Gr—Greece, A—Austria, Sl—Slovenia.

Moreover, Figure 5 showed that analyzed data from 2021 had similar profiles and have been described by restrictions and health system policies groups. Similarly, data from 2022 also had similar profiles but predominantly has been described by vaccination and new cases groups as well as the presence of the Omicron variant. 

## 4. Discussion

### 4.1. Findings

Our cross-sectional study shows that analyzed countries in the EU (Austria, Greece, Germany, Italy and Slovenia) had similar profiles in 2021 based on data from weeks 22 to 30, which were characterized by restrictions and health system policies, as well as SARS-CoV-2 variants, such as B.1.351, B.1.1.7, B.1.617.2 and P.1. Similarly, these countries had similar profiles in 2022 in weeks 22 to 30. However, in 2022, the countries characterized by other variables, such as number of new COVID-19 cases per million, number of people fully vaccinated per hundred, number of total boosters administered per hundred and also occurrence of Omicron variant with sub-lineages. Moreover, BA.5 sub-lineage was dominant in analyzed period in 2022 year.

Based on examined data, the analyzed period of 2021 was characterized by a higher level of restrictions compared to the same period of 2022. Since the start of pandemic, many countries in Europe implemented restrictions and national lockdowns, which led to a decrease in the mobility and contact of people, and thus inhibited the COVID-19 infections [21]. The restrictions, such as school closures, international travel restrictions and public gathering bans had the most influence on the COVID-19 pandemic [22]. Our analysis showed similar results: the profiles of analyzed countries in 2021 may be the best characterized by closed public events, restrictions on gatherings, international travel controls and a higher value of stringency index. Moreover, the countries had differences in the speed and scale of introducing restrictions; in most European countries, testing and isolating of all infected people were delayed, except in Germany, in contrast to most Asian countries. Moreover, in Asian countries, most of the infected people were isolated at institutions, not at home. Due to the fact that previous pandemics, SARS (severe acute respiratory syndrome) and MERS (Middle East respiratory syndrome), caused by the other coronaviruses, have occurred in Asia, these countries were better prepared for a new epidemic, and the population was also better prepared for strong restrictions [23]. A study conducted on 300 women showed that during the pandemic, handwashing and disinfection of hands using sanitizer were increased [24]. To inhibit the spread of COVID-19, the usage of face masks or respirators was also recommended. In King County in the U.S., the observational study showed that in general people agree to mask wearing in public areas, especially adults aged over 60 years [25]. Obligatory mask wearing has proven to be successful in limiting the virus spreading, as a study conducted in California reported that wearing a mask indoors in public places decreased the risk of COVID-19 infection [26].

On the other hand, the profiles of analyzed countries were changed in 2022. Although the number of fully vaccinated people as well as those vaccinated with booster doses is high, there is also an increase in the number of infected people, which is confirmed by our analysis. The question is whether only the new variant of SARS-CoV-2 contributes to the increased incidence of COVID-19 during this period or is it affected by a low level of government restrictions? It is also unknown if the vaccines are effective against the Omicron variant, including sub-lineages. There is evidence that vaccines are reasonably effective against this variant of the virus. During the proxy Omicron period in South Africa, the efficacy of two doses of BNT162b2 vaccine equaled 70% compared to 93% in the period with Delta variant domination [27]. Moreover, a study from Canada shows that three doses of mRNA vaccines with different schematic vaccination protocols had similar effectiveness against COVID-19 infection with the Omicron variant: all three doses of BNT162b2 vaccine had an effectiveness of 32%, while three doses of mRNA-1273 vaccine had 44%, and two doses of BNT162b2 vaccine with booster dose of mRNA-1273 vaccine had 36% [28]. Similar results were reported from Qatar: the estimated effectiveness of BNT162b2 booster dose was 49.4%, while mRNA-1273 was 47.3% against symptomatic Omicron COVID-19 cases [29]. After 20 weeks, vaccination with two doses of ChAdOx1 nCoV-19 had no effect on the Omicron variant, whereas the booster doses of BNT162b2 or mRNA-1273 vaccines increase the efficacy to 62.4% or 70.1%, respectively. On the other hand, the efficacy of BNT162b2 primary course was 8.8% from 25 weeks, but it was increased to 67.2% after the BNT162b2 booster dose and 73.9% after the mRNA-1273 booster dose. However, the efficacy after booster doses diminished over time [30].

### 4.2. Research Limitations

Although our study contains significant results for understanding the factors that influence the COVID-19 pandemic, it also includes some limitations. An important problem with the use of data stored in publicly available repositories is if some data are missing, which may be related to submitting data from countries at different times. Hence, the problem of delayed data renewal in databases. Another problem is that not all COVID-19 cases are sequenced to identify SARS-CoV-2 variants. According to the SARS-CoV-2 variants dashboard [31], among analyzed countries, in Austria, during the period from 22 to 30 weeks in 2021, around 70–80% of cases were sequenced, whereas at the same period in 2022 the figure was only 19–61.9%. The situation is worse in Germany, where 11–16.5% of cases were sequenced in 2021, but around 1% in 2022. Similar, in Italy in 2021, 5–25.9% of cases were sequenced and 0.1–1% in 2022, whereas in Greece 5.7–19.8% of cases were sequenced in 2021, but merely 0.3–1.2% in 2022. However, in Slovenia, in 2021, 20.7–94% of cases were sequenced, while the percent of sequenced cases in 2022 had not been reported. Despite the abovementioned limitations to our study, it highlights the most important factors affecting the COVID-19 pandemic in the European Union in summer 2022.

## 5. Conclusions

Despite the high COVID-19 vaccination rate in the European Union, the daily number of new cases increases in most countries during the investigated period of time. In our study, we showed that the vaccination rate is relatively high during the summer of 2022, simultaneously, and the increased number of COVID-19 cases seems to be connected with the presence of the Omicron variant and its sub-lineages. There is no doubt that vaccination followed by booster doses enhances the effectiveness of COVID-19 vaccination against the constantly mutating SARS-CoV-2 virus. These may be used by governments in vaccination strategies.

## Data Availability

Publicly available datasets were analyzed in this study. This data can be found here: [http://www.worldometers.info/ (accessed on 4 August 2022); https://ourworldindata.org (accessed on 17 August 2022); https://www.ecdc.europa.eu/en (accessed on 17 August 2022); https://www.eurocontrol.int (accessed on 17 August 2022); https://www.bsg.ox.ac.uk/ (accessed on 17 August 2022)].
